# Elevated Carbon Dioxide and Chronic Warming Together Decrease Nitrogen Uptake Rate, Net Translocation, and Assimilation in Tomato

**DOI:** 10.3390/plants10040722

**Published:** 2021-04-08

**Authors:** Dileepa M. Jayawardena, Scott A. Heckathorn, Krishani K. Rajanayake, Jennifer K. Boldt, Dragan Isailovic

**Affiliations:** 1 Department of Environmental Sciences, University of Toledo, Toledo, OH 43606, USA; dileepa.jayawardena@utoledo.edu; 2 Department of Chemistry and Biochemistry, University of Toledo, Toledo, OH 43606, USA; krishani.rajanayake@utoledo.edu (K.K.R.); dragan.isailovic@utoledo.edu (D.I.); 3 U.S. Department of Agriculture, Agricultural Research Service, Toledo, OH 43606, USA; jennifer.boldt@usda.gov

**Keywords:** climate change, elevated CO_2_, heat stress, nitrogen assimilation, nitrogen metabolism, nitrogen uptake, *Solanum*, tomato, warming

## Abstract

The response of plant N relations to the combination of elevated CO_2_ (eCO_2_) and warming are poorly understood. To study this, tomato (*Solanum lycopersicum*) plants were grown at 400 or 700 ppm CO_2_ and 33/28 or 38/33 °C (day/night), and their soil was labeled with ^15^NO_3_^−^ or ^15^NH_4_^+^. Plant dry mass, root N-uptake rate, root-to-shoot net N translocation, whole-plant N assimilation, and root resource availability (%C, %N, total nonstructural carbohydrates) were measured. Relative to eCO_2_ or warming alone, eCO_2_ + warming decreased growth, NO_3_^−^ and NH_4_^+^-uptake rates, root-to-shoot net N translocation, and whole-plant N assimilation. Decreased N assimilation with eCO_2_ + warming was driven mostly by inhibition of NO_3_^−^ assimilation, and was not associated with root resource limitations or damage to N-assimilatory proteins. Previously, we showed in tomato that eCO_2_ + warming decreases the concentration of N-uptake and -assimilatory proteins in roots, and dramatically increases leaf angle, which decreases whole-plant light capture and, hence, photosynthesis and growth. Thus, decreases in N uptake and assimilation with eCO_2_ + warming in tomato are likely due to reduced plant N demand.

## 1. Introduction

Anthropogenic CO_2_ and other greenhouse gas emissions have increased significantly since industrialization, warming the planet. Global climate models predict atmospheric CO_2_ concentration will be about 420 to 935 ppm, and global mean surface temperature will increase by about 1.4 to 5.8 °C, by the end of this century [1,2]. This rise in temperature may cause both acute and chronic heat stress in plants, affecting both root and shoot functions [3]. Although CO_2_ enrichment alone benefits plants (e.g., increased photosynthesis and water-use efficiency), these beneficial effects may disappear when eCO_2_ is compounded with other climate-change variables, such as supra-optimal temperatures [4,5,6,7]. For example, the combination of eCO_2_ and warming decreased growth and root N-uptake rate in tomato, relative to either factor alone [5]. In addition, eCO_2_ alone is not always beneficial to plants, as it can result in a dilution of tissue N concentration (%N) due to increased photosynthetic assimilation of C, resulting in plant tissue of lower nutritional quality for food [8,9].

Since plants procure and assimilate nutrients through nutrient-specific-uptake and -assimilatory proteins, crop improvement under future climate conditions could be achieved by modification of these proteins using transgenic or genetic engineering, or traditional plant breeding approaches, the latter by identifying species or genotypes with better-adapted nutrient-uptake and -assimilation mechanisms [8]. However, in order to implement such efforts, we should identify which biochemical pathway (e.g., uptake vs. translocation vs. assimilation) and which biochemical component (e.g., uptake proteins vs. assimilatory proteins) to target. Nitrogen is often the most limiting nutrient for plants [10]. Plants can procure N either in inorganic or organic forms, but most plants procure the majority of their N primarily as inorganic N (NO_3_^−^ and NH_4_^+^) [11]. A number of studies have investigated the effects of eCO_2_, or warming alone, on plant N uptake, N translocation, and N assimilation, but studies examining the combined effects of eCO_2_ and warming on these responses are scarce.

Though eCO_2_ tends to decrease root N-uptake rate (N uptake per unit mass or length), past studies show that root N-uptake rate in response to eCO_2_ can be highly variable and can depend on the form of N supplied [9,12,13]. Optimum temperature for plant growth and function is species specific, and the effect of warming on root N uptake depends on whether the temperature increase is from suboptimal to optimal or from optimal to supra-optimal. Warming from suboptimal to optimal often increases root N-uptake rate [14,15,16,17], while warming from optimal to supra-optimal (acute or chronic) often decreases root N uptake [17,18,19,20,21]. The limited evidence available suggests that the interactive effects of eCO_2_ and warming on N uptake can be equivocal. Coleman and Bazzaz [22], using *Abutilon theophrasti*, and Jayawardena et al. [5], using *Solanum lycopersicum*, showed that the interactive effect of eCO_2_ plus warming can inhibit root N-uptake rate in C_3_ plants when compared with other treatments (i.e., eCO_2_ or warming alone). In addition, in the C_4_ species, *Amaranthus retroflexus*, N-uptake rates varied with plant ages in response to eCO_2_ plus warming [22]. Using ^15^N labeling, Arndal et al. [23] found no effect of eCO_2_ plus warming on NO_3_^−^ nor NH_4_^+^-uptake rates of *Calluna vulgaris* (an evergreen dwarf shrub) and *Deschampsia flexuosa* (a perennial grass). Dijkstra et al. [24] also found no interactive effect of eCO_2_ and warming on NO_3_^−^ uptake of grasses in a semiarid grassland.

Studies that investigated root-to-shoot N translocation in response to eCO_2_ showed or suggested a consistent decreasing trend with eCO_2_ [25,26,27,28,29]. As Cohen et al. [26] explained, one potential reason for decreased N translocation in response to eCO_2_ could be the reduced size of xylem volume when plants are grown at eCO_2_. Nitrogen translocation from roots to shoots in response to temperature has been studied in some detail, but results were highly variable. In most studies, the highest temperature examined was 30 °C or less, and the temperature was altered only in the root-zone while maintaining the shoots at a control temperature [15,30,31,32,33,34]. These studies showed that increased root-zone temperature can increase [15,31,32], decrease [32,34], or have no effect [33] on, root-to-shoot N translocation. Moreover, a study conducted by Hungria and Kaschuk [35] showed that whole-plant heat stress (39 vs. 28 or 34 °C) can reduce xylem organic-N translocation in *Phaseolus vulgaris*, while Mainali et al. [21] suggested whole-plant acute heat stress (40 vs. 30 °C) did not affect N translocation in *Andropogon geradii*. As with N-uptake rate, data on root-to-shoot N translocation in response to eCO_2_ plus warming are scarce. Rufty et al. [31] studied the interactive effect of root-zone temperature (18, 24, and 30 °C) and eCO_2_ (1000 vs. 400 ppm) on N translocation of *Glycine max* supplied NO_3_^−^ as the sole N source, and they noted an increase with eCO_2_ plus root warming. In contrast, based on lower transpiration and leaf ^15^N isotopic composition observed in *Triticum durum* at eCO_2_ (700 vs. 400 ppm) plus warming (ambient vs. ambient + 4 °C), Jauregui et al. [4] concluded that eCO_2_ plus warming can reduce root-to-shoot N translocation.

Plant N assimilation in response to eCO_2_ has been extensively studied. A number of studies have shown that eCO_2_ can inhibit shoot NO_3_^−^, but not NH_4_^+^, assimilation in C_3_ plants [36,37,38,39]. However, challenging this view, Andrews et al. [40] showed that eCO_2_ does not inhibit NO_3_^−^ assimilation in C_3_ plants, and the assimilation of both forms of N take place in a similar way in response to eCO_2_. In response, Bloom et al. [41] stated that eCO_2_ inhibits shoot NO_3_^−^ assimilation, but enhances NO_3_^−^ assimilation in roots of C_3_ plants. This is consistent with results of Jauregui et al. [27], who suggested that eCO_2_ favored N assimilation in roots over shoots in *T. durum*, based on the low shoot-to-root NR activity ratio observed at eCO_2_. As with N uptake, one could expect N assimilation to increase as temperature rises from suboptimal-to-optimal, and decrease with optimal-to-supra-optimal temperatures, because N assimilation is carried out by enzymes which have temperature optima. The majority of studies that have investigated temperature effects on shoot N assimilation have looked at the effect of heat stress on NR activity only, and they reveal that NR activity diminished with heat stress [35,42,43,44]. Using two *Agrostis* species, Rachmilevitch et al. [45] investigated the effects of root-zone temperature (37 vs. 20 °C) on the rate of plant NO_3_^−^ assimilation, and noted it decreased with increased temperature. The limited evidence from these studies suggests that optimal to supra-optimal temperature increases are most likely to reduce plant N or NO_3_^−^ assimilation. Nitrogen assimilation is a key process that influences the nutritional quality of food and, recently, researchers have started investigating how it responds to eCO_2_ plus warming. To date, three reports showed a decreasing trend for N assimilation in response to eCO_2_ plus warming. Vicente et al. [46] investigated the effects of eCO_2_ (700 vs. 370 ppm) and warming (ambient vs. ambient + 4 °C) at two levels of N supply on C and N metabolism of *T. durum*, using gene expression analysis. Based on decreased soluble protein, amino acids, and NR activity in flag leaves, they showed that N assimilation can be inhibited by eCO_2_ and warming. Since most of the genes involved in N metabolism are post-transcriptionally or post-translationally regulated [10,47], gene expression measurements alone do not necessarily reflect phenotypic effects on N metabolism. A study conducted by Jauregui et al. [4] also reported that eCO_2_ (700 vs. 400 ppm) plus warming (ambient + 4 °C) inhibited N assimilation in flag leaves of *T. durum*, based on decreased levels of amino acids, total soluble protein, and NR activity. Root N assimilation in response to eCO_2_ (700 vs. 400 ppm) plus chronic warming (37 vs. 30 °C) was indirectly investigated by Jayawardena et al. [5] in *S. lycopersicum*, and they suggested eCO_2_ plus warming can inhibit root N assimilation, which could have resulted from the observed decreases in levels of N-assimilatory proteins that were measured in roots. Notably, none of the previous studies investigated whole-plant N assimilation in response to eCO_2_ plus warming.

The aforementioned review reveals a lack of studies have investigated the combined influence of eCO_2_ and chronic warming on plant N metabolism. Therefore, the objective of this study was to determine the individual and interactive effects of eCO_2_ and chronic warming on NO_3_^−^ and NH_4_^+^ uptake rates, net N translocation, and whole-plant N and NO_3_^−^ assimilation, using tomato (*S. lycopersicum*) as a model. The information resulting from this study will be helpful for crop scientists, plant breeders, and molecular biologists to understand how N metabolism of tomato and other plants will respond to future climates, and how to develop new tomato genotypes with improved N use under future climate conditions.

## 2. Results

**Figure 1:**
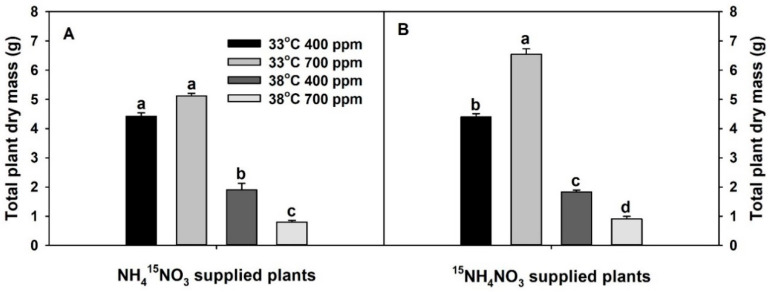
Effects of ambient (400 ppm) vs. elevated (700 ppm) CO_2_ and near-optimal (33 °C) vs. chronic warming (38 °C) day-time temperatures on total plant dry mass (g) of *Solanum lycopersicum* labeled for 3 days with 1 mM (**A**) NH_4_^15^NO_3_ or (**B**) ^15^NH_4_NO_3_ and grown for 21 or 23 days, respectively. Each bar represents mean (*n* = 5) + 1 standard error of mean (SEM). Within each panel, bars not sharing the same letters are significantly different (*p* < 0.05, Tukey’s test).

Total plant dry mass was significantly decreased with chronic warming (Table S1, Figure 1). In contrast, the effect of eCO_2_ on plant dry mass was dependent on the treatment temperature. Elevated CO_2_ significantly and non-significantly increased the plant dry mass of ^15^NH_4_^+^-supplied and ^15^NO_3_^−^-supplied plants at 33 °C, respectively, while it significantly decreased the plant dry mass of both sets of plants at 38 °C. Plants grown at eCO_2_ plus chronic warming had the lowest dry mass.

**Figure 2:**
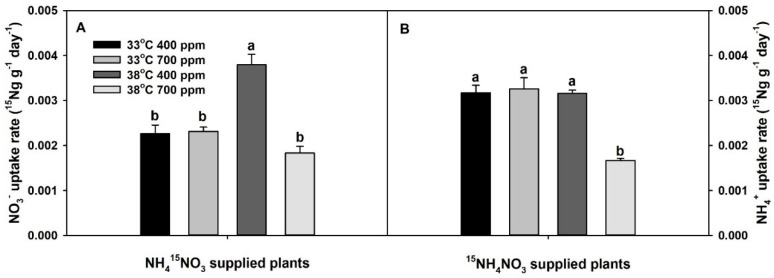
Effects of ambient (400 ppm) vs. elevated (700 ppm) CO_2_ and near-optimal (33 °C) vs. chronic warming (38 °C) day-time temperatures on (**A**) NO_3_^−^ uptake rate (^15^Ng g^−1^ day^−1^) and (B) NH_4_^+^ uptake rate (^15^Ng g^−1^ day^−1^) of *Solanum lycopersicum* labeled for 3 days with 1 mM (**A**) NH_4_^15^NO_3_ or (**B**) ^15^NH_4_NO_3_ and grown for 21 or 23 days, respectively. Each bar represents mean (*n* = 5) + 1 SEM. Within each panel, bars not sharing the same letters are significantly different (*p* < 0.05, Tukey’s test).

Both NO_3_^−^ and NH_4_^+^-uptake rates were significantly affected by the interaction of CO_2_ × temperature (Table S1). Though chronic warming significantly increased NO_3_^−^-uptake rate at ambient CO_2_ (aCO_2_), it did not influence NH_4_^+^-uptake rate at aCO_2_. Elevated CO_2_ did not influence either NO_3_^−^ or NH_4_^+^-uptake rates at 33 °C, but it tended to decrease NO_3_^−^-uptake rate and significantly decreased NH_4_^+^-uptake rate at 38 °C (relative to 33 °C and aCO_2_). Hence, as with total plant dry mass, plants grown at eCO_2_ plus chronic warming had the lowest NO_3_^−^ and NH_4_^+^-uptake rates (Figure 2). Notably, the NH_4_^+^-uptake rate was greater than the NO_3_^−^-uptake rate at 33 °C, regardless of the CO_2_ treatment (approximately ×1.4). However, when the temperature increased from 33 °C to 38 °C, NO_3_^−^-uptake rate surpassed the NH_4_^+^-uptake rate, regardless of the CO_2_ treatment (approximately ×1.1–1.2).

**Figure 3:**
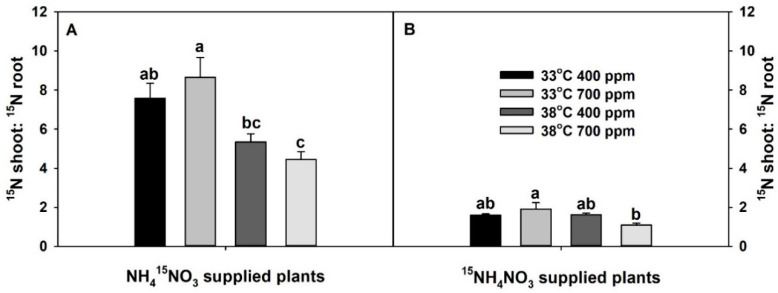
Effects of ambient (400 ppm) vs. elevated (700 ppm) CO_2_ and near-optimal (33 °C) vs. chronic warming (38 °C) day-time temperatures on the ratio of total ^15^N content in the shoots vs. roots of *Solanum lycopersicum* labeled for 3 days with 1 mM (**A**) NH_4_^15^NO_3_ or (**B**) ^15^NH_4_NO_3_ and grown for 21 or 23 days, respectively. Each bar represents mean (*n* = 5) + 1 SEM. Within each panel, bars not sharing the same letters are significantly different (*p* < 0.05, Tukey’s test).

The ratio of total ^15^N content in the shoots vs. roots of ^15^NO_3_^−^-supplied plants was significantly affected only by the temperature, while that of ^15^NH_4_^+^-supplied plants was significantly affected by both temperature and the interaction of CO_2_ × temperature (Table S1). Chronic warming tended to decrease the ratio of total ^15^N content in the shoots vs. roots of ^15^NO_3_^−^-supplied plants at aCO_2_. However, it did not influence the ratio of ^15^NH_4_^+^-supplied plants at aCO_2_. Though eCO_2_ tended to increase the ratio of total ^15^N content in the shoots vs. roots of plants treated with isotopes of both N forms at 33 °C, it significantly or non-significantly decreased the ratio of total ^15^N content in the shoots vs. roots in both ^15^NO_3_^−^ and ^15^NH_4_^+^-supplied plants at 38 °C. As with plant dry mass and N-uptake rate, plants grown at eCO_2_ plus chronic warming had the lowest ratio of total ^15^N content in the shoots vs. roots (Figure 3). The ratio of total NO_3_^−^ in shoots vs. roots was significantly affected by both individual and interactive effects of CO_2_ and temperature, while the ratio of NH_4_^+^ in shoots vs. roots was affected only by CO_2_ (Figure S1). Elevated CO_2_ significantly increased shoot:root NO_3_^−^ ratio at 33 °C, but not at 38 °C (Figure S1A). Elevated CO_2_ marginally decreased shoot:root NH_4_^+^ ratio at both temperatures (Figure S1B).

**Figure 4:**
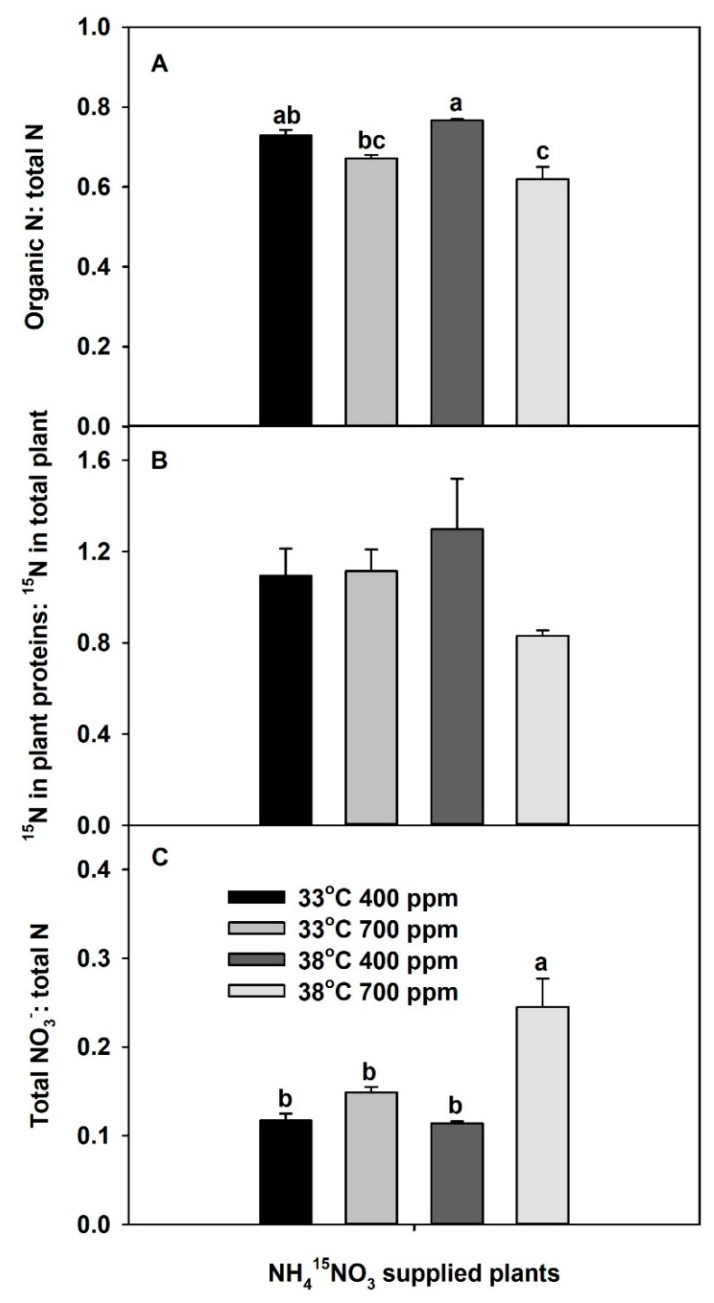
Effects of ambient (400 ppm) vs. elevated (700 ppm) CO_2_ and near-optimal (33 °C) vs. chronic warming (38 °C) day-time temperatures on (**A**) organic N: total N, (**B**) ^15^N in plant proteins: ^15^N in total plant, and (**C**) total NO_3_^−^: total N ratios of *Solanum lycopersicum*, labeled for 3 days with 1 mM NH_4_^15^NO_3_ and grown for 21 days. Each bar represents mean (*n* = 5) + 1 SEM. Within each panel, bars not sharing the same letters are significantly different (*p* < 0.05, Tukey’s test); no letters are shown if there are no differences.

The ratio between organic N to total N was significantly affected by both CO_2_ and the interaction of CO_2_ × temperature (Table S1). Elevated CO_2_ tended to decrease the organic-N:total-N ratio at 33 °C (Figure 4A). In contrast, the effect of chronic warming on the ratio between organic N to total N was dependent on the CO_2_ treatment; chronic warming tended to increase the ratio at aCO_2_, while it significantly decreased the ratio at eCO_2_. Though the ratio between total ^15^N in plant proteins to total ^15^N in the plant of ^15^NO_3_^−^-supplied plants was not significantly affected by CO_2_ and/or temperature (Table S1), it responded in a similar way as the ratio between organic N to total N in response to the independent variables (Figure 4B). Furthermore, plants grown at eCO_2_ plus chronic warming had the lowest ratio of ^15^N in total plant protein to ^15^N in the total plant. In addition, the ratio between total plant NO_3_^−^ to total plant N was greatest in the plants grown at eCO_2_ plus chronic warming (Figure 4C). Neither eCO_2_ at 33 °C nor chronic warming at aCO_2_ affected this ratio significantly, relative to aCO_2_ and 33 °C. However, eCO_2_ significantly increased the ratio between total plant NO_3_^−^ to total plant N at 38 °C.

**Figure 5:**
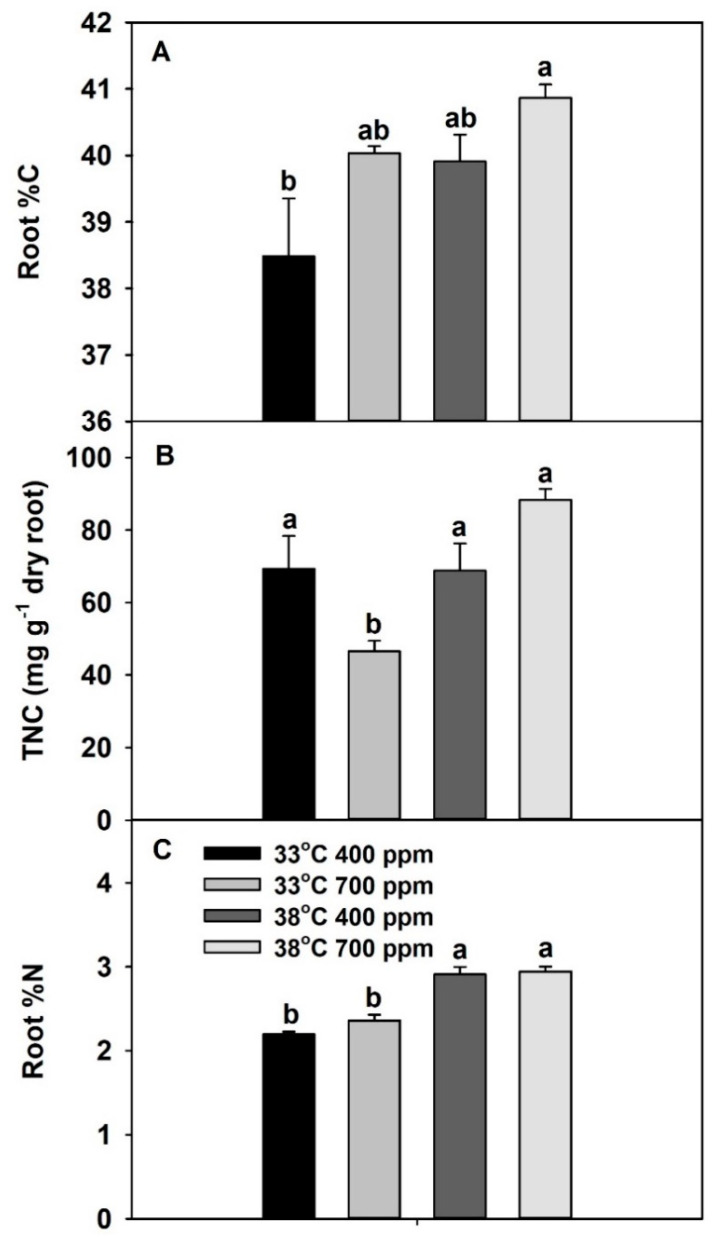
Effects of ambient (400 ppm) vs. elevated (700 ppm) CO_2_ and near-optimal (33 °C) vs. chronic warming (38 °C) day-time temperatures on (**A**) root %C, (**B**) total nonstructural carbohydrates (mg g^−1^ dry root mass), and (**C**) root %N of *Solanum lycopersicum*, grown for 21 days. Each bar represents mean (*n* = 5) + 1 SEM. Within each panel, bars not sharing the same letters are significantly different (*p* < 0.05, Tukey’s test).

Root %C was significantly affected by both temperature and CO_2_, while root total nonstructural carbohydrate (TNC) was significantly affected by temperature and the interaction of temperature × CO_2_ (Table S1). Notably, both root %C and TNC tended to increase with the combination of eCO_2_ plus chronic warming, compared with the other treatments (Figure 5A,B). Root %N was significantly higher at 38 vs. 33 °C, while CO_2_ had no significant effect on root %N (Figure 5C, Table S1).

**Figure 6:**
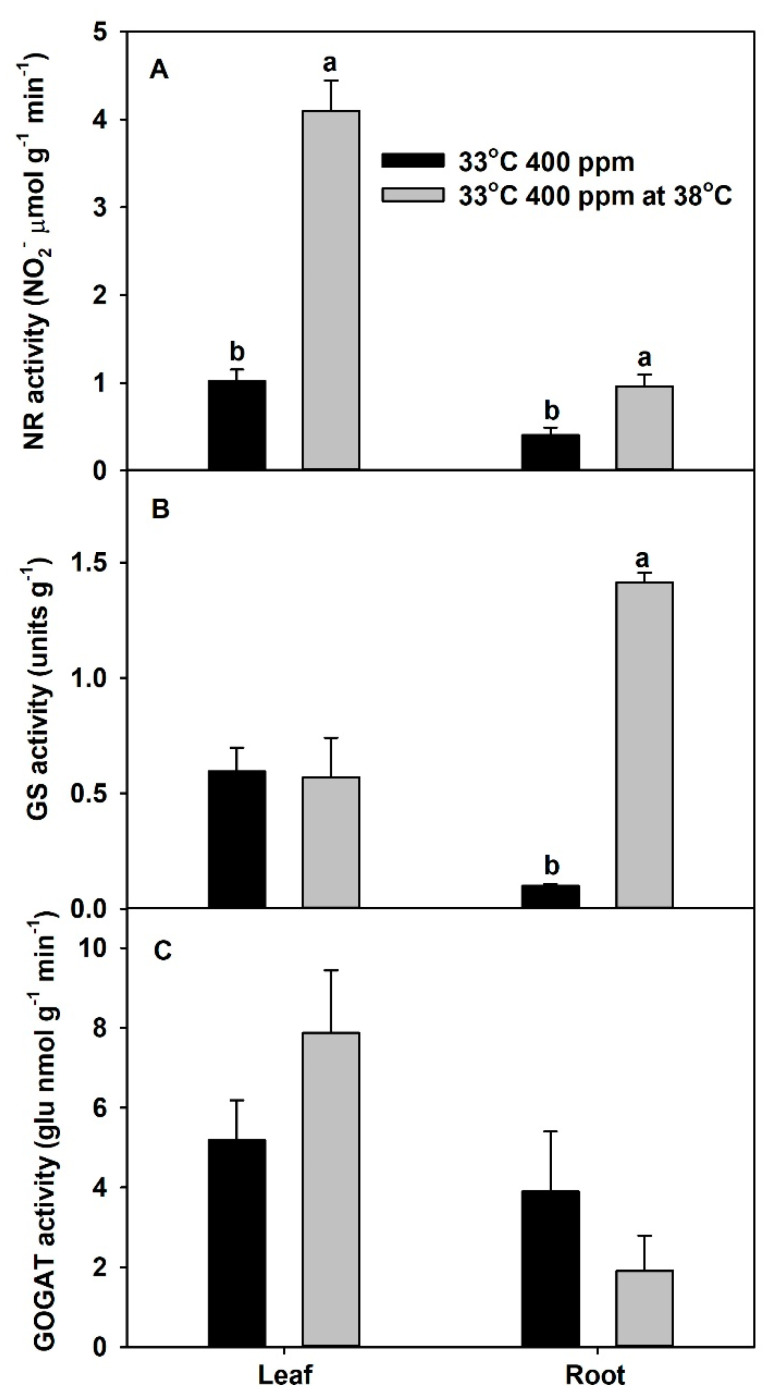
In vitro activities of (**A**) nitrate reductase (NR), (**B**) glutamine synthetase (GS), and (**C**) glutamine oxoglutarate aminotransferase (GOGAT), extracted from leaves or roots of *Solanum lycopersicum* plants grown at 33 °C and 400 ppm and measured at both 33 and 38 °C. Each bar represents mean (*n* = 4) + 1 SEM. Within each panel, bars not sharing the same letters are significantly different (*p* < 0.05, Tukey’s test); no letters are shown if no differences.

In vitro activity (a measure of maximum potential activity) of NR in both leaves and roots was significantly increased at 38 °C vs. 33 °C. In addition, the in vitro activity of GS in roots also significantly increased at 38 °C. However, the in vitro activities of leaf GS and leaf and root GOGAT were unaffected by the higher temperature of 38 °C (Figure 6).

## 3. Discussion

To date, most previous studies have focused on single-factor manipulation approaches when investigating the effects of global environmental changes (e.g., CO_2_ enrichment, warming, drought, and N deposition) on plant N relations. However, as we expect these changes to occur concurrently in the future, multifactor manipulation approaches will be necessary to understand the impacts of climate change on plants. The combined effects of CO_2_ enrichment and warming on plant N relations (uptake, translocation, and assimilation) were investigated in this study for this reason. As in our previous studies [5,48], the combination of eCO_2_ plus chronic warming severely inhibited the growth of tomato, relative to either factor alone. Plants grown at eCO_2_ plus chronic warming had the lowest N-uptake rate, net N translocation, and N assimilation when compared with plants grown at eCO_2_ or chronic warming alone.

In general, it is expected that eCO_2_ will increase plant biomass and supra-optimal temperatures (heat stress) will decrease plant biomass when water and nutrients are not limiting; this general trend was also observed in this study. However, the limited evidence from past studies shows that the interactive effects of eCO_2_ plus warming on plant biomass can be highly variable. Some studies have shown eCO_2_ plus warming to have a neutral or positive effect on plant growth [49], while others have shown eCO_2_ plus warming to have a negative effect [5,6,7,48]. Our growth reduction can be partly explained by decreased light interception of leaves, and, thus, in situ photosynthesis, caused by a vertical growth orientation of leaves (hyponasty) that occurs when tomato is grown under eCO_2_ plus warming [48]. The mechanism for eCO_2_ + warming leaf hyponasty is not known, but, so far, appears to be restricted to compound-leaved species and is especially dramatic in tomato and potato. We have examined several tomato genotypes so far (hybrids and heirlooms, all indeterminant), and this hyponasty response occurs in all genotypes (with minor variation) [48].

The effect of eCO_2_ on plant N uptake has not been consistent [12]. This is likely partly due to differences among studies in experimental protocols (e.g., length of CO_2_ exposure, assaying intact vs. excised roots, differences in N source or amount) and partly due to naturally occurring variations among species [12]. At least two possible mechanisms can explain how eCO_2_ can affect N uptake: (1) in the short term, eCO_2_ increases plant growth and, hence, plant N demand. This, in turn, increases N-uptake capacity [12], and (2) eCO_2_ induces stomatal closure and this decreases the transpiration-driven mass flow of N, which, in turn, decreases N uptake by roots [9,50]. However, in this study, eCO_2_ did not affect either NO_3_^−^ or NH_4_^+^-uptake rates at near-optimal growth temperature, but it did decrease uptake rates of both at 38 °C (and the effect eCO_2_ + warming was non-additive). Further, plants grown at eCO_2_ plus chronic warming had the lowest rates of NO_3_^−^ and NH_4_^+^ uptake. Previously, we used sequential harvesting (vs. ^15^N labeling in this study) to show that the combination of eCO_2_ (700 vs. 400 ppm) plus chronic warming (37 vs. 30 °C) can reduce root N-uptake rate of plants treated with either NO_3_^−^ or NH_4_^+^ singly (not NH_4_NO_3_ as in this study) [5]. This earlier study further showed that decreases in N-uptake rate may be due, at least in part, to decreased concentration or activity of N-uptake proteins (NRT1 and AMT1). Notably, chronic warming significantly increased NO_3_^−^-uptake rate, but it did not influence NH_4_^+^-uptake rate at aCO_2_. An enhanced rate of NO_3_^−^ uptake with chronic warming could be due to the stimulation of uptake kinetics [12], and the neutral effect of chronic warming on NH_4_^+^-uptake rate could be to reduce excess accumulation of NH_4_^+^ and avoid NH_4_^+^ toxicity [16]. Moreover, Bassirirad [12] showed that the NH_4_^+^:NO_3_^−^-uptake ratio depends on soil or root temperature, and this ratio decreased as soil or root temperature increased from suboptimal to optimal levels (sufficient data were lacking to examine whether this statement holds true for optimal to supra-optimal temperature rises). Our results indicate this statement holds true even when temperature increases from optimal to supra-optimal levels. At 33 °C, NH_4_^+^-uptake rate was approximately 1.4 times greater than NO_3_^−^-uptake rate, regardless of the CO_2_ treatment. However, when temperature increased from 33 °C to 38 °C, NO_3_^−^-uptake rate increased to approximately 1.1–1.2 times that of NH_4_^+^-uptake rate, regardless of the CO_2_ treatment.

The ratio of total ^15^N in the shoots vs. the roots was used as a proxy for net N translocation from roots to shoots. Here, we were careful to use the term “net N translocation” instead of “N translocation”, as N can continuously circulate between roots and shoots. For example, foliar feeding of leaves with ^15^NO_3_^−^ has shown that leaf NO_3_^−^ can be translocated to every part of the plant, including the root system [51]. Warm-season species, such as tomato, prefer shoot over root NO_3_^−^ assimilation, so most of the soil-derived NO_3_^−^ is translocated from roots to shoots and assimilated there [52]. This is consistent with the high shoot-to-root ^15^N ratios (>4) for ^15^NO_3_^−^-supplied plants in all four treatments in this study. Since NH_4_^+^ assimilation generates H^+^, and shoots have a limited capacity for proton disposal, nearly all soil-derived NH_4_^+^ is assimilated in roots [53]. Again, our results (low shoot-to-root ^15^N ratio of <2) are consistent with limited translocation of NH_4_^+^ from roots to shoots in all four treatments. Although eCO_2_ did not influence NO_3_^−^ or NH_4_^+^-uptake rates, it tended to increase net N translocation of both ^15^NO_3_^−^ and ^15^NH_4_^+^ in plants at 33 °C. This caused a significant increase in the ratio of total NO_3_^−^ in shoots-to-roots (Figure S1A). The ratio of NH_4_^+^ content in shoot-to-roots was significantly affected only by CO_2_ (*p* = 0.0285), and there was a trend for slightly lower shoot:root NH_4_^+^ with eCO_2_ regardless of the temperature (Figure S1B). Though we did not measure photorespiration in this study, the slight decrease in shoot:root NH_4_^+^ ratio with eCO_2_ could be due to an inhibition of photorespiration by eCO_2_. In C_3_ plant leaves, NH_4_^+^ flux from photorespiration is 5 to 10-fold higher than that from NO_3_^−^ reduction [47]. Since limited soil-derived NH_4_^+^ is typically translocated from roots to shoots, a higher percentage of shoot NH_4_^+^ is likely represented by NH_4_^+^ derived from photorespiration. Though chronic warming significantly increased NO_3_^−^-uptake rate, it tended to decrease net N translocation in ^15^NO_3_^−^-supplied plants at aCO_2_. This resulted in a low shoot:root NO_3_^−^ ratio (Figure S1A). As with NO_3_^−^ and NH_4_^+^ uptake rate, the interactive effect of eCO_2_ plus chronic warming caused a decrease in net N translocation from roots-to-shoots in both ^15^NO_3_^−^ and ^15^NH_4_^+^-supplied plants. This also resulted in a low shoot:root NO_3_^−^ ratio in plants grown at eCO_2_ plus warming (Figure S1A).

The two ratios, total-plant organic N to total N, and total-plant ^15^N in proteins to total-plant ^15^N, were used as proxies for whole-plant N assimilation (decreases of these ratios denote inhibition), while the total NO_3_^−^:total N ratio was used as a proxy for whole-plant NO_3_^−^ assimilation (increases of this ratio denote inhibition). Previous studies showed that eCO_2_ can inhibit shoot NO_3_^−^ assimilation and enhance root NO_3_^−^ assimilation [38,41]. In this study, eCO_2_ did not influence the ratio of ^15^N in proteins to total plant, but it decreased the organic N to total N ratio at 33 °C, indicating some inhibition of plant N assimilation. Moreover, total NO_3_^−^:total N ratio was marginally increased with eCO_2_ at 33 °C, which also indicates that eCO_2_ may have a tendency to inhibit total-plant NO_3_^−^ assimilation. In contrast, chronic warming may slightly increase both organic N:total N and protein ^15^N:plant ^15^N at aCO_2_, indicating a possible marginal stimulation of total-plant N assimilation. Chronic warming did not have an effect on total NO_3_:total N ratio at aCO_2_. However, these plants had the lowest total NH_4_^+^:total N ratio (data not shown). Therefore, the slight stimulation of N assimilation by chronic warming at aCO_2_ could have been due to the stimulation of NH_4_^+^ rather than NO_3_^−^ assimilation. The combined effect of eCO_2_ and chronic warming decreased both organic N:total N (significantly) and plant ^15^N:total ^15^N (marginally), indicating an inhibition of total-plant N assimilation. Moreover, eCO_2_ plus chronic warming significantly increased total NO_3_^−^:total N ratio, indicating an inhibition of NO_3_^−^ assimilation by eCO_2_ plus chronic warming. Our results suggest the inhibition of whole-plant N assimilation by eCO_2_ plus chronic warming was mainly due to inhibition of whole-plant NO_3_^−^ assimilation. However, based on root %N and total-root protein data, Jayawardena et al. [5] suggested that eCO_2_ plus chronic warming can inhibit root N assimilation in plants provided only NO_3_^−^ or only NH_4_^+^. They further showed that, when plants were grown at eCO_2_ plus warming, the roots had decreased levels of N-assimilatory proteins per gram root (i.e., nitrate reductase, NR, glutamine synthetase, GS, and glutamine oxoglutarate aminotransferase, GOGAT). Based on those results, we can assume that the inhibition of total-plant N assimilation by eCO_2_ plus chronic warming could be due to decreased levels of these N assimilatory proteins. Further, we assessed the in vitro activities of assimilatory proteins extracted from plants grown at 33 °C, at both 33 and 38 °C. For all three proteins, the activities at 38 °C were greater or similar to 33 °C (Figure S2), which confirms that these assimilatory proteins are not damaged by chronic warming temperature.

Previously, using wheat as the model species, Jauregui et al. [4] reported that eCO_2_ (700 vs. 400 ppm) plus warming (ambient + 4 °C) reduced leaf N assimilation by reducing energy availability. Since plants grown at eCO_2_ plus chronic warming had the lowest N-uptake rates and N assimilation, we hypothesized that this could be due to the lower energy or resource availability in roots to perform root functions, which we tested by measuring root %C, TNC, and %N. Relative to the other treatments; plants grown at eCO_2_ plus warming had the highest root %C and TNC concentrations, while they also had the co-highest root %N. Based on these results, we concluded that the low rates of N uptake and N assimilation in plants grown at eCO_2_ plus warming were not due to limited energy or resource availability in roots.

In summary, tomato plants grown at eCO_2_ plus chronic warming had the lowest plant dry mass, NO_3_^−^ and NH_4_^+^-uptake rates, net N translocation, and whole-plant N (and NO_3_^−^) assimilation when compared with other treatments. Previously, we showed that N uptake can be inhibited by eCO_2_ (700 vs. 400 ppm) plus warming (37 vs. 30 °C), using a sequential harvesting technique [5]; furthermore, in this study, we showed that both NO_3_^−^ and NH_4_^+^-uptake rates can be inhibited by eCO_2_ plus warming using ^15^N labeling. Moreover, in this study, we showed that net N translocation can be inhibited by eCO_2_ plus warming using two different methods: (1) the ^15^N ratio between shoots and roots, and (2) the NO_3_^−^ ratio between shoots and roots. Finally, we showed that the whole-plant N assimilation can be inhibited by eCO_2_ plus warming using two methods: (1) the ratio between organic N and total N, and (2) the ratio between ^15^N in proteins and ^15^N in the plant). Inhibition of whole-plant N assimilation was mainly due to the inhibition of NO_3_^−^ assimilation by eCO_2_ plus chronic warming. In addition, the decreased rates of N uptake and N assimilation were not due to the resource limitation (N or C) for root functions, but, probably, due to the decreased levels of enzymes involved in N metabolism (NR, GS, GOGAT), as shown previously [5]. Overall, this study has shown that the interactive effects of CO_2_ enrichment and global warming can negatively affect plant N metabolism in tomato, which will have serious consequences for the production and nutrient quality of tomato, one of the world’s most-important non-grain food crops. Given global human population is expected increase by 1.4 to 3.9 billion by 2050, global crop production will need to increase by then by ca. 70% to meet global food demand [54]. This study provides valuable information regarding weak links in N metabolism in response to CO_2_ enrichment and global warming that can be targeted for improvement, in order to improve yield and nutrient quality of tomato, and, perhaps, other crops in the future.

## 4. Materials and Methods

### 4.1. Plant Material, Growth Conditions, and Treatments

Tomato (*S. lycopersicum* L. cv. Big Boy), a heat-tolerant warm-season C_3_ vegetable crop, was used as the model species. Seeds were germinated in trays filled with calcined clay in a greenhouse and watered daily. In the greenhouse, temperature fluctuated between 25–30 °C, and supplementary lighting (<15% of full sunlight) was provided by 250-W high-pressure sodium (GE Lighting Inc., Cleveland, OH, USA) and 400-W metal-halide (Osram Sylvania Products Inc., Manchester, NH, USA) lamps to provide a 15-h photoperiod. When seeds were germinating, quarter-strength complete nutrient solution was added to the trays only after cotyledons began turning yellow (nutrient concentrations of the full-strength solution: 2 mM MgSO_4_, 1 mM KH_2_PO_4_, 1 mM K_2_HPO_4_, 2 mM CaCl_2_, 71 µM Fe-DTPA, 10 µM MnCl_2_, 50 µM H_3_BO_3_, 6 µM CuSO_4_, 6 µM ZnSO_4_, 1 µM Na_2_MoO_4_, and 1 mM NH_4_NO_3_; pH = 6.0). The nutrient solution used in this study provided N at an intermediate but limiting amount, and was designed to follow up on a previous study of ours examining effects of nitrate vs. ammonium [5], but the nutrient solution provided other nutrients at near-optimal levels e.g., [53,55,56]. When seedlings were at the first-adult-leaf stage, uniform seedlings were transplanted into 3.1-L cylindrical pots [10-cm diameter × 40-cm length PVC pipes; one plant per pot] filled with a mixture of calcined clay and perlite in a 3:1 (*v*:*v*) ratio (supported by mesh at the bottom to hold the substrate).

After transplant, all pots were transferred to four growth chambers (model E36HO, Percival Scientific Inc., Perry, IA, USA). Chambers were initially programmed to have the near-optimal growth temperature for this cultivar (33/28 °C day/night air temperatures), ambient CO_2_ (400 ppm), ambient humidity (ca. 55–65%), 600 µmol m^−2^ s^−1^ PAR (supplied by 55-W Osram Dulux luminous lamps; Osram GmbH, Augsburg, Germany), and a 14-h (0600–2000 h) photoperiod. Each chamber received 14 pots; 10 were used for either ^15^NO_3_^−^ or ^15^NH_4_^+^ treatments (*n* = 5) while the other 4 were used as unlabeled controls. Pots were placed in individual shallow trays to retain any excess water and nutrient solution. Plants were kept inside the growth chambers for three days to acclimate to the new environment. During this period, quarter-strength complete nutrient solution (500 mL) was added to each pot once. When plants were free from transplant-stress and at their second adult-leaf stage, the temperature of the high-temperature-treatment chambers was increased gradually from near-optimal to chronic warming temperature (38/33 °C day/night) over three days to avoid potential heat shock. The temperatures used in this study were based on previous experiments that showed that optimal daytime temperature for this cultivar in our hands is 32–33 °C, as far as whole plant biomass of pre-reproductive plants, and that significant reductions in biomass only occur at temperatures of 36–38 °C [5,20,48]. Once the high-temperature-treatment chambers reached the chronic warming temperature, CO_2_ treatment was started (day 0). A 2 × 2 factorial experimental design was used, with two levels of CO_2_ [ambient (400 ppm) vs. elevated (700 ppm)] and two temperature treatments (33/28 °C vs. 38/33 °C day/night), with one growth chamber per treatment. Plants were fertilized with 500 mL of full-strength complete nutrient solution containing 2 mM N (1 mM NH_4_NO_3_) every other day. Plants were rotated within each chamber every 4–5 days to avoid potential position/edge effects and switched between chambers every 7–8 days to avoid potential chamber effects.

On day 18, a randomly selected subset of plants from each chamber (*n* = 5) was labeled with 1950 mL (this volume is equivalent to the estimated soil pore-volume in the 3.1-L pots) of full-strength complete nutrient solution containing 1 mM NH_4_^15^NO_3_. On day 19, another randomly selected subset of plants (unlabeled controls, *n* = 4) from each chamber was treated with 1950 mL of nutrient solution containing 1 mM (unlabeled) NH_4_NO_3_. On day 20, the remaining plants (*n* = 5) were labeled with 1950 mL of nutrient solution containing 1 mM ^15^NH_4_NO_3_. The nutrient solution was isotopically labeled by adding either NH_4_^15^NO_3_ or ^15^NH_4_NO_3_ with an isotopic purity of 98 atom % ^15^N (Sigma-Aldrich Inc, St. Louis, MO). When making the isotopically labeled nutrient solutions, carbenicillin (an antibiotic) was added (at 20 mg L^−1^) to avoid external nitrification, de-nitrification, and N immobilization by microbes, as in [57]. Addition of carbenicillin did not affect plant growth (based on preliminary experiments). Plants were harvested three days (based on preliminary experiments) after ^15^N isotope labeling (day 21 for ^15^NO_3_^−^-labeled plants and day 23 for ^15^NH_4_^+^-labeled plants). Unlabeled plants were harvested on day 22, and these were used to determine background levels of ^15^N in plant tissue. At the time of harvest, all plants were in the vegetative stage. This labeling experiment was repeated at a later time, with results from the two experiments being nearly identical, and results are presented for the first experiment only.

### 4.2. Plant N and Protein Analysis

Harvested plants were separated into shoots and roots, and then each shoot and root system was divided into two halves and weighed separately. One half of the shoots and roots were flash frozen in liquid N_2_ and stored at −80 °C to be used for tissue NH_4_^+^ and protein analysis (fresh tissue). The other half of the shoots and roots were put in paper bags separately and placed in an oven at 70 °C for 72 h. Dry mass of shoots and roots were measured using these samples (the ratio between dry and fresh mass of these samples were used to calculate total shoot and root dry mass).

Dried shoot and root samples were pulverized separately using a mortar and pestle. A subset of each pulverized sample was used to measure shoot and root %C and %N using the combustion-MS technique [58]. Another subset of each pulverized sample was analyzed to measure ^15^N (atom%) in solid samples using an elemental analyzer interfaced to a continuous-flow isotope-ratio mass spectrometer at the University of California, Davis, stable-isotope facility. Nitrate or NH_4_^+^-uptake rates were calculated as the total amount of ^15^N taken up by plants treated with ^15^NO_3_^−^ or ^15^NH_4_^+^ per g of dry root per day (using root mass at the final harvest). Background ^15^N was subtracted using ^15^N (atom%) of unlabeled plants. Net N translocation was calculated as the ratio of total ^15^N content in the shoots vs. roots of ^15^NO_3_^−^ or ^15^NH_4_^+^-supplied plants.

Since NO_3_^−^ is the main source of N for most crop species [59], we studied the responses of ^15^NO_3_^−^ supplied plants in more detail. Therefore, shoot and root NO_3_^−^ concentrations were measured only from NH_4_^15^NO_3_-supplied plants, using the colorimetry-based salicylic-acid method, as explained in [60]. Briefly, 50 mg of pulverized tissue was suspended in 5 mL of deionized water, incubated at 45 °C for 1 h, and centrifuged at 10,000× *g* for 5 min. The supernatant was recovered and 0.2 mL of the extracted NO_3_^−^ sample was reacted with 0.8 mL of 5% (*w*/*v*) salicylic acid–sulfuric solution and 19 mL of 2 N NaOH. Absorbance was measured at 410 nm using KNO_3_^−^ as the standard. The NO_3_^−^ (total pool size) ratio between shoots and roots was used as a metric of net NO_3_^−^ translocation. Shoot and root NH_4_^+^ from NH_4_^15^NO_3_ supplied plants were quantified using an NH_4_^+^ ion-selective electrode (Hach Co., Loveland, CO). Briefly, fresh tissue was ground to a fine powder using liquid N_2_, mortar, and pestle. Then, 500 mg of tissue was suspended in a tube containing 30 mL of 0.001 M CaSO_4_ solution (pH = 3) [61]. The tube was shaken for 30 min in a shaker and then centrifuged at 4000× *g* for 10 min [62]. The recovered supernatant was used for NH_4_^+^ quantification. The NH_4_^+^ (total pool size) ratio between shoots and roots was used as a metric of net-NH_4_^+^ translocation. Total-plant inorganic N content was estimated as the sum of total-NO_3_^−^ and -NH_4_^+^ contents. Total-plant organic N content was estimated as the difference between total-plant N and total-plant inorganic N. The ratio between organic N and total N was used as a metric of total-plant N assimilation. The ratio between total NO_3_^−^ and total N was used as a metric of total-plant NO_3_^−^ assimilation.

Shoot and root proteins were extracted from fresh tissues of NH_4_^15^NO_3_ supplied plants, as described in [5]. Briefly, a known amount of fresh shoot or root tissue was ground in liquid N_2_ to a fine powder, then, with a known volume of extraction buffer (EB). The resulting mixture was transferred to a tube, an equal volume of buffer-saturated phenol was added, and the tube was centrifuged at 10,000× *g* for 10 min. Protein in the phenol phase was further purified by centrifugation after adding an equal volume of EB to the recovered phenol phase. Extracted protein was precipitated by adding five volumes of chilled 0.1 M ammonium acetate to the recovered phenol phase. Protein was pelleted down by centrifugation. The resulting pellet was washed several times with ammonium acetate and acetone. Following this method, proteins were extracted on two sets of samples per biological replicate. In the first extraction, protein pellets were freeze dried in a lyophilizer (Genesis 25 SQ Super ES; VirTis-SP Scientific, Gardiner, NY, USA) and analyzed to measure ^15^N (atom%) in protein samples (as above). In the second extraction, protein pellets were dissolved in a resolubilizing buffer. Then, total shoot or root protein concentration per g of fresh shoot or root was measured using a colorimetric assay (DC protein assay; Bio-Rad Laboratories Inc., Hercules, CA, USA), using bovine serum albumin (BSA) as the protein standard. Using data on ^15^N (atom%) in protein pellets, protein concentration (mg g^−1^ dry mass), ^15^N (atom%) in oven-dried samples, and dry mass data, the ratio between ^15^N in plant proteins and ^15^N in total plant (pool size) was calculated as another metric of plant N assimilation.

### 4.3. Total Nonstructural Carbohydrate Assay

Total nonstructural carbohydrate concentration in roots was measured according to the method described by Chow and Landhäusser [63], with some modifications. Briefly, 1 mL of 0.1 N NaOH was added to 25 mg of pulverized dried tissue in a tube, incubated at 50 °C for 30 min, and then 1 mL of 0.1 N HCl was added. Then, 1 mL of an enzyme solution containing 3000 units/mL of α-amylase, plus 15 units/mL amyloglucosidase in 0.05 M sodium acetate, were added to the tube, and then tubes were incubated at 50 °C for 24 h. The digest was filtered through 0.22 µm polyvinylidene difluoride (PVDF) filters to remove digestion enzymes. The filtrate (0.5 mL) was reacted with 1 mL of 2% phenol and 2.5 mL of H_2_SO_4_, incubated at room temperature for 30 min, and absorbance was measured at 490 nm using glucose to generate a standard curve.

### 4.4. In Vitro N-Assimilatory-Protein Activity Assays

In an independent experiment, tomato (*S. lycopersicum* L. cv. Big Boy) seeds were germinated in a greenhouse, transplanted to pots, transferred to a growth chamber, and treated with ambient CO_2_ and near-optimal temperature, as above. Chambers contained 12 pots for NR, GS, and GOGAT shoot and root in vitro activity assays (*n* = 4). Plants were grown for 19–23 days, but without isotope labeling. The third fully expanded leaf below the apical meristem was harvested for leaf NR, GS, or GOGAT activity assays, while the first 5–10 cm from the root tip was harvested for root NR, GS, or GOGAT activity assays. For all three enzymes, assays were performed as soon as the tissues were harvested (within 30 min).

Nitrate reductase activity was measured according to the protocol described by Sigma Aldrich for EC 1.6.6.1. A known amount of fresh tissue (0.5 g) was ground with 2 mL of reagent A (25 mM KH_2_PO_4_, 10 mM KNO_3_, 0.05 ethylenediaminetetraacetic acid (EDTA), 0.5% polyvinylpolypyrrolidone (PVPP), 1 mM phenylmethylsulfonyl fluoride (PMSF), 1 mM leupeptin, and 3% (*w*/*v*) BSA). The mixture was transferred to a tube and centrifuged at 12,100× *g* for 5 min at 4 °C, and the supernatant (with enzyme) was recovered. In two other tubes, 1.8 mL of reagent A was mixed with 100 µL of 2 mM β-NADH and equilibrated at 33 °C or 38 °C temperature. The enzyme reaction was started by adding 100 µL of the enzyme mixture to each tube. Then, the reaction was stopped by adding 1 mL of 58 mM sulfanilamide in 3 M HCl at time = 0 min (tube 1) and after 5 min (tube 2). Finally, 1 mL of 0.77 mM N-(1-naphthyl) ethylenediamine dihydrochloride was added to each tube and absorbance was measured at 540 nm using NaNO_2_ to generate a standard curve. In vitro NR activity was calculated as NO_2_^−^ produced per g of tissue per minute.

Glutamine synthetase activity was measured according to the protocol described by Sigma Aldrich for EC 6.3.1.2. A reaction cocktail (pH = 7.1) was prepared by mixing 100 mM imidazole, 3 M sodium glutamate, 250 mM ATP, 900 mM MgCl_2_, 1 M KCl, and 1.2 M NH_4_Cl. A known amount of fresh tissue (0.5 g) was ground with 2 mL of an EB (50 mM Tris-HCl (pH = 7.1), 1 mM EDTA, 0.6% (*w*/*v*) PVPP, 1 mM PMSF, and 1 mM leupeptin). The mixture was transferred into a tube, centrifuged at 12,100× *g* for 10 min at 4 °C, and the supernatant (with enzyme) was recovered. In another tube, 2.7 mL of the reaction cocktail was mixed with 100 µL of 33 mM phosphoenol pyruvate (PEP), 60 µL of 12.8 mM β-NADH, and 40 µL of pyruvate kinase/L-lactic dehydrogenase enzyme mixture, and equilibrated at treatment temperature (33 °C or 38 °C). The first enzyme mixture (100 µL) was added to the second reaction mixture, and then the decrease in the absorbance at 340 nm (A_340_) for 10 min was recorded. Using the maximum–slope linear range of the absorbance-time relationship, ΔA_340_/min was calculated, after subtracting for background absorbance.

Glutamine oxoglutarate aminotransferase activity was measured according to the method described by Berteli et al. [64] and Lutts et al. [65]. A known amount of fresh tissue (0.5 g) was ground with 2 mL of an EB (100 mM KH_2_PO_4_, 0.05 mM EDTA, 0.5% (*w*/*v*) PVPP, 1 mM PMSF, 1 mM leupeptin, and 2 mM 2-oxoglutarate), the mixture was transferred into a tube and centrifuged at 12,100× *g* for 15 min at 4 °C, and then the supernatant (with enzyme) was recovered. Next, 100 µL of the enzyme mixture was mixed in a tube with 1.8 mL of a reaction mixture containing 125 mM phosphate buffer (pH = 7.5), 10 mM 2-oxoglutarate, 10 mM glutamine, 10 mM aminoxy-acetate, and 10 mM methyl viologen and equilibrated at treatment temperature (33 °C or 38 °C). The reaction was started by adding 100 µL of a mixture containing 4.7 µg of sodium dithionite and 5 µg of NaHCO_3_. The tube was incubated at treatment temperature for 30 min. The reaction was stopped by boiling the tube in a water bath for 5 min. The tube was centrifuged at 5000 rpm for 5 min, and then the supernatant was recovered. In the supernatant, glutamate was separated from glutamine by anion-exchange chromatography. During anion-exchange chromatography, the column (Pasteur pipette) was packed with the anion-exchange resin, AG1X8 (acetate form), to a length of 4 cm and gradually washed with 5 mL of HPLC-grade water. Then, 2 mL of the recovered supernatant was passed through the column. The column was again washed with 12 mL of HPLC-grade water and glutamate was eluted with 8 mL of 1 M acetic acid [66]. To purify the glutamate, the eluent was completely dried using a vacuum evaporator at room temperature. The dried glutamate samples were redissolved, and the concentration was determined by electrospray ionization-mass spectrometry (ESI-MS, Orbitrap Fushion Tribid Mass Spectrometer, Thermo Fisher, Waltham, MA, USA) using sodium glutamate as the standard.

### 4.5. Statistical Analysis

The statistical software, RStudio version 3.6.1 [RCore Team (2013), Vienna, Austria], was used for all statistical analyses. Statistical assumptions of independence, normality, and equal variance were checked with residual vs. fitted, normal Q-Q, and S-L plots, respectively. If statistical assumptions were not met, data were transformed; log transformation proved to be sufficient. Data were analyzed using two-way (two levels of CO_2_ × two levels of temperature) analysis of variance (ANOVA), with CO_2_ and temperature as fixed factors. Results were considered significant if *p* < 0.05. If ANOVA results were significant, a Tukey’s post hoc test was performed for multiple comparisons. Figures were generated using SigmaPlot version 14.0 (Systat Software, Inc., San Jose, CA, USA). Results presented in figures are untransformed means and standard errors of mean (SEM).

## Data Availability

All data are available on request.

## Supplementary Materials

The following are available online at https://www.mdpi.com/article/10.3390/plants10040722/s1. Table S1: Results from ANOVA statistical analysis. Figure S1: Effects of ambient (400 ppm) vs. elevated (700 ppm) CO_2_ and near-optimal (33 °C) vs. chronic warming (38 °C) day-time temperatures on (A) shoot: root NO_3_^−^ and (B) shoot to root NH_4_^+^ ratios of *Solanum lycopersicum*, grown for 21 days. Each bar represents mean (*n* = 5) + 1 SEM. Within each panel, bars not sharing the same letters are significantly different (*p* < 0.05, Tukey’s test); no letters are shown if no differences.
