# Supplementary material for: Elevated Carbon Dioxide and Chronic Warming Together Decrease Nitrogen Uptake Rate, Net Translocation, and Assimilation in Tomato
Source: Plants (Basel). 2021 Apr 8;10(4):722. doi: 10.3390/plants10040722 (PMC8067974; doi:10.3390/plants10040722)
Supplement: Supplementary file 1 [file plants-10-00722-s001.pdf]

Table S1. Results from ANOVA statistical analysis.

	Response variable	Temperature	CO_2	Temperature × CO_2
$NH_4^{15}NO_3$ supplied plants	Total plant dry mass	<0.001	<0.001	<0.001
	NO_3^- uptake rate	0.0672	<0.001	<0.001
	^{15}N shoots: ^{15}N roots ratio	<0.001	0.8937	0.1778
	Organic N: total N ratio	0.6803	<0.001	0.0194
	^{15}N in plant proteins: ^{15}N in plant ratio	0.3849	0.0811	0.0565
	Total NO_3^-: total N ratio	0.0134	0.0002	0.0091
	Root %C	0.0356	0.0217	0.5562
	Total non-structural carbohydrates	0.0010	0.6715	0.0015
	Root %N	<0.001	0.1480	0.3330
$^{15}NH_4NO_3$ supplied plants	Total plant dry mass	<0.001	0.0058	<0.001
	NH_4^+ uptake rate	<0.001	<0.001	<0.001
	^{15}N shoots: ^{15}N roots ratio	0.0400	0.5445	0.0307

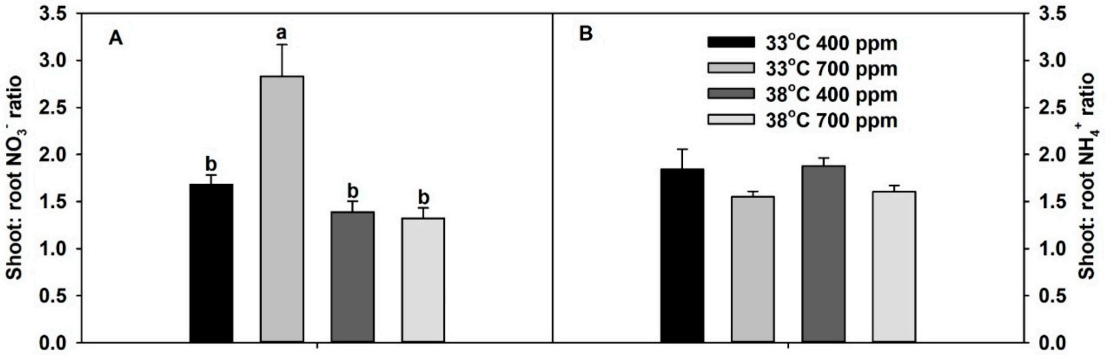

Figure S1. Effects of ambient (400 ppm) vs. elevated (700 ppm) CO_2 and near-optimal (33°C) vs. chronic warming (38°C) day-time temperatures on (A) shoot: root NO_3^- and (B) shoot to root NH_4^+ ratios of *Solanum lycopersicum*, grown for 21 days. Each bar represents mean (n = 5) + 1 SEM. Within each panel, bars not sharing the same letters are significantly different (P < 0.05, Tukey's test); no letters are shown if no differences.
